# Comparison of wrist-worn Fitbit Flex and waist-worn ActiGraph for measuring steps in free-living adults

**DOI:** 10.1371/journal.pone.0172535

**Published:** 2017-02-24

**Authors:** Anne H. Y. Chu, Sheryl H. X. Ng, Mahsa Paknezhad, Alvaro Gauterin, David Koh, Michael S. Brown, Falk Müller-Riemenschneider

**Affiliations:** 1 Saw Swee Hock School of Public Health, National University of Singapore, Singapore, Singapore; 2 Department of Computer Science, School of Computing, National University of Singapore, Singapore, Singapore; 3 PAPRSB Institute of Health Sciences, Universiti Brunei Darussalam, Jalan Tungku Link, Gadong, Brunei Darussalam; 4 Department of Electrical Engineering and Computer Science, Lassonde School of Engineering, York University, Toronto, Ontario, Canada; 5 Institute of Social Medicine, Epidemiology and Health Economics, Charité University Medical Centre Berlin, Berlin, Germany; Vanderbilt University, UNITED STATES

## Abstract

**Introduction:**

Accelerometers are commonly used to assess physical activity. Consumer activity trackers have become increasingly popular today, such as the Fitbit. This study aimed to compare the average number of steps per day using the wrist-worn Fitbit Flex and waist-worn ActiGraph (wGT3X-BT) in free-living conditions.

**Methods:**

104 adult participants (n = 35 males; n = 69 females) were asked to wear a Fitbit Flex and an ActiGraph concurrently for 7 days. Daily step counts were used to classify inactive (<10,000 steps) and active (≥10,000 steps) days, which is one of the commonly used physical activity guidelines to maintain health. Proportion of agreement between physical activity categorizations from ActiGraph and Fitbit Flex was assessed. Statistical analyses included Spearman’s rho, intraclass correlation (ICC), median absolute percentage error (MAPE), Kappa statistics, and Bland-Altman plots. Analyses were performed among all participants, by each step-defined daily physical activity category and gender.

**Results:**

The median average steps/day recorded by Fitbit Flex and ActiGraph were 10193 and 8812, respectively. Strong positive correlations and agreement were found for all participants, both genders, as well as daily physical activity categories (Spearman's rho: 0.76–0.91; ICC: 0.73–0.87). The MAPE was: 15.5% (95% confidence interval [CI]: 5.8–28.1%) for overall steps, 16.9% (6.8–30.3%) vs. 15.1% (4.5–27.3%) in males and females, and 20.4% (8.7–35.9%) vs. 9.6% (1.0–18.4%) during inactive days and active days. Bland-Altman plot indicated a median overestimation of 1300 steps/day by the Fitbit Flex in all participants. Fitbit Flex and ActiGraph respectively classified 51.5% and 37.5% of the days as active (Kappa: 0.66).

**Conclusions:**

There were high correlations and agreement in steps between Fitbit Flex and ActiGraph. However, findings suggested discrepancies in steps between devices. This imposed a challenge that needs to be considered when using Fibit Flex in research and health promotion programs.

## Introduction

New wearable technologies have helped raise individual self-awareness about physical activity behavior. Among all the functionalities that a range of wearable devices have, step counting is the most fundamental and consistently found feature. Step counts have been proposed as a health indicator for population studies [1], and even community-based health-promotion programs [2]. The 10,000 steps/day guideline is one of the commonly used physical activity indices [3]. Various government/professional organizations around the world have used the 10,000 daily steps recommendation as an index of high physical activity level. This daily step-based recommendation has been endorsed by the World Health Organization (WHO), National Heart Association of Australia, US Centers for Disease Control and Prevention, and American Heart Association to improve overall health. For healthy adults, it appears that this guideline is a realistic estimate of an appropriate daily physical activity level [4, 5]. It was suggested that those achieving the goal of 10,000 steps per day were more likely to meet physical activity guidelines as compared to those with lower step counts [2]. Furthermore, health promotion programs that included a daily step goal were reportedly more successful in increasing physical activity than those without this component [6]. The use of step data (usually as steps/day) is a simple means of reflecting habitual physical activity pattern, and this approach has become acceptable to many researchers and practitioners [1, 6]. Moreover, walking activity has been reported as a prevalent form of leisure-time physical activity and a functional task in the daily lives [7].

Among all the accelerometers commonly used in research, the ActiGraph (Pensacola, FL, USA) is well-validated and has been extensively used for assessing physical activity under free-living conditions [8–11]; The ActiGraph accelerometers use algorithms to quantify and contextualize the resultant acceleration signals of human motion. They have shown high accuracy for moderate-to-high walking speed stepping in the laboratory (compared to direct observations, ICC: 0.72–0.99) and under free-living conditions (compared to the Yamax Digiwalker, ICC: 0.90) [12]. The ActiGraph has been used in large-scale epidemiological studies such as the US National Health and Nutrition Examination Survey (NHANES) [13], and the Women’s Health Study (WHS) [14].

Recently, consumer-based activity trackers (e.g. Fitbit, Jawbone UP, LUMOback, Nike+ Fuelband, Omron Walking Style Pro pedometer, etc.) and in-built accelerometers in smartphones have become increasingly popular [15, 16]. It was forecasted that the smart wearables market could reach 170 million units by 2017 [17]. Fitbit (San Francisco, CA, USA) is one of the most commonly used brands amongst the consumer-based activity trackers. As of 2015, Fitbit had reached 9.5 million active users [18]. Among their products, the wrist-worn Fitbit Flex has become popular in recent years either for aesthetic reasons or wearing comfort. The Fitbit Flex is sleek and displays only LED with a tap screen. Users are able to monitor and access data on the number of steps, sleep quality, and other personal metrics through the Fitbit dashboard. This could be useful for targeted physical activity interventions designed to achieve healthy behaviors. It was suggested that wrist-worn accelerometers allowed for monitoring of low-intensity activities, and were associated with considerable increases in wearing compliance and data quality [19].

A number of studies have validated wireless consumer-based monitors of different brands in measuring step counts and energy expenditure [16, 20–23]. A recent systematic review concluded high validity for the Fitbit Classic, One and Zip compared to accelerometry-based step counts (particularly in laboratory settings) [24]. It was further highlighted that more field-based studies are needed. Evaluation of the trackers in assessing free-living physical activity (non-controlled environment outside a lab setting) is particularly important, as the results are more likely to reflect usual day-to-day activities. To date, sample sizes of studies on the Fitbit Flex validity under free-living conditions have been relatively small (ranging from 14 to 25 participants) and based on young adults [16, 25–27]. Of note, one similar study was limited by a small sample size of one adult only [28]. However, despite the high correlation between activity trackers, these studies generally showed that Fitbit Flex has measurement limitations regarding the overestimation and underestimation of activity levels compared with the reference device, depending on different study settings and types of activity [26, 27].

Given these considerations and highlighted gaps, this study aimed to make standardized comparisons based on step counts from the consumer-oriented Fitbit Flex and the research-grade ActiGraph wGT3X-BT. Differences in levels and types of physical activity between males and females have been reported [29, 30]. It was reported that more males than females tended to practise sports (e.g. soccer, basketball, etc.), whereas females were more likely to engage in yoga, dancing, aerobics, etc. [31]. Because these differences may influence their accuracy in measurement, we further performed gender specific analysis. Hence, the objectives of this study were:

1. To compare free-living steps/day recorded by the Fitbit Flex and the ActiGraph wGT3X-BT accelerometers in all participants, by each step-defined daily physical activity category and gender.
2. To compare the agreement between devices in classifying participants’ step-defined daily physical activity categories.

## Materials and methods

### Study design and participants

This was a cross-sectional study. The present study was a part of a previously published study [32], whereby a convenience sample of 107 employees who completed both ActiGraph and Fitbit Flex measures were included. Participants from a large public University and a hospital in Singapore were recruited between February 2014 and June 2014. Individuals were residing in Singapore and were of various ethnicities (Chinese, Malay, Indian and others). Participants were invited to take part in this study through mass e-mailing. Individuals who indicated interest were approached and interviewed by the researcher.

The inclusion criteria were:

1. Males and females aged 21 to 65 years
2. Either students or working adults
3. Absence of physical disabilities or illness that would create abnormal gait patterns.

The study was approved by the National University of Singapore Institutional Review Board (NUS-IRB Ref No.: B-14-021). Participants provided their written informed consent to participate in this study.

### Procedure

The goals and procedures of the study were explained to each participant by the researcher via face-to-face interview. Participants’ information on gender, age, education level, height and weight were self-reported. Instructions were given to the participants by trained personnel on how to put on a wrist-worn Fitbit Flex and a waist-worn ActiGraph concurrently for 7 days. Instruction manuals on the proper use of the ActiGraph and Fitbit Flex were also given to participants for additional guidance. Participants were instructed that the devices had to be worn for at least 10 hours/day, and could be removed at night depending on their comfort level. They were asked to complete a daily time sheet to record each wearing day when both devices were worn while maintaining their normal activities. Information required on the time sheet comprised of the dates they started and stopped wearing the devices.

### ActiGraph wGT3X-BT

The ActiGraph™ wGT3X-BT monitor (ActiGraph, LLC, Pensacola, Florida, USA) is a triaxial accelerometer (Dimensions: 4.6cm x 3.3cm x 1.5cm; weight: 19 grams) worn on the waist using an elastic belt to secure above the right hip bone for quantifying the amount and frequency of human movements. The monitor was initialized at a sample rate of 30Hz to record activities for free-living conditions. Participants were instructed to wear the ActiGraph for 7-day. They were allowed to remove the ActiGraph only while bathing or immersing the body in water. ActiGraph data were downloaded using ActiLife 6 software (ActiGraph, LLC, Pensacola, FL, USA) by the researchers upon collection of the devices. Downloaded data were integrated into 60-sec epochs.

### Fitbit Flex

Fitbit Flex^TM^ (Dimensions: 22.2cm x 6.0cm x 6.0cm; weight: 100 grams) is a wrist-worn wearable wireless sensor with a triaxial accelerometer that records physical activity throughout the day. It can sync with a smartphone application/computer. Participants were instructed to wear the Fitbit Flex on their non-dominant wrist, for the same duration as the ActiGraph (up to 7-day) concurrently. In general, Fitbit Flex requires the creation of individual user accounts to download stored data using a Web-based software application. However, for the purpose of our study, anonymous user accounts were created by the study team which could only be accessed by the researchers. Steps data were therefore stored on the devices, and the minute-by-minute Fitbit Flex data were downloaded at the end of each participant’s wearing period by the study team.

### Data reduction

For wear time validation, because the ActiGraph accelerometer is an established device to measure physical activity with many validation studies determining their accuracy [33, 34], valid wear time determined by the ActiGraph was regarded as the reference. A detailed description of the procedures on ActiGraph wear time validation and removal of sleep time can be found elsewhere [32]. Then, a valid day was defined as having an accumulation of ≥1500 steps/day with ≥10 hours/day restricted only to common wear time based on both ActiGraph and Fitbit Flex. The 1500 steps/day criterion was based on a previous research conducted by Tudor-Locke et al. comparing accelerometers positioned at different locations under free-living conditions [35]. All participants with ≥4 valid days of data were included in the analysis. Additionally, wear time was also verified based on the daily time sheets.

### Statistical analysis

All statistical procedures were performed using SPSS software (version 20.0). The significance level was set at *P*<0.05. Descriptive characteristics were presented as mean (standard deviation; SD) or median (interquartile range; IQR). Shapiro-Wilk test was used to determine whether the data was normally distributed. Differences in the characteristics between genders were detected by non-parametric tests. Mann-Whitney U test (for continuous variables), chi-squared test (for categorical variables) and Fisher’s exact test (for categorical variables with cells having an expected frequency of five or less) were used.

Analyses of the relationship between ActiGraph and Fitbit Flex were performed across: all participants, by each category of step-defined daily physical activity, and gender. Because there could be potential within-subject variations, comparison of step counts for the magnitude of relationship between the two devices was done on a day-to-day basis. Spearman’s correlation coefficient (rho) and intraclass correlation coefficient (ICC) were used to assess correlation and agreement, respectively in steps between ActiGraph and Fitbit Flex. An ICC value of ≥0.75 implied excellent, 0.60–0.74 good, 0.40–0.59 fair and <0.40 poor agreement [36]. Median of absolute percentage error (MAPE) between devices was calculated: (absolute error/observed steps) × 100%. The difference in MAPE by each category of step-defined daily physical activity and gender was compared using Mann-Whitney U test. ActiGraph derived steps/day was used to classify two step-defined activity categories for the assessments of Spearman’s rho and ICC. The classification of days into two step-defined activity categories was adapted based on previous studies: valid days with a cumulative of ≥10,000 steps/day were considered as active days, and <10,000 steps/day were inactive days [5, 37, 38]. As for the Bland-Altman analysis, a non-parametric approach was adopted since the differences between the two devices were non-normally distributed. Bland-Altman plots were presented as median, 10^th^ and 90^th^ percentiles to display variance around differences between two devices. Proportion of agreement in achievement of 10,000 steps per day produced by ActiGraph and Fitbit Flex was assessed using Kappa.

## Results

Out of 107 recruited participants, 104 were included because they met the wear time criteria and provided 682 days of data. Table 1 shows participants' sociodemographic characteristics of the study. Participants had a median age of 31.0 years (IQR: 26.0–42.8), predominantly female (66.3%), and had a university degree (74.0%). On average, 6.6 valid wear days were recorded per participant and there was no significant difference between males and females. The ActiGraph and Fitbit Flex steps were significantly higher in males than females (*P* = 0.03 and 0.01 for ActiGraph and Fitbit Flex, respectively).

**Table 1:** Characteristics of study population.

		All (n = 104)	Males (n = 35)	Females (n = 69)	*P-*value^a^
Age (Med; IQR)		31.0; 26.0–42.8	33.0; 27.0–50.0	30.0; 25.5–40.5	0.05
Height, cm (Med; IQR)		163.0; 157.0–169.8	170.0; 168.0–175.0	160.0; 155.0–163.0	<0.001
Weight, kg (Med; IQR)		60.0; 53.0–69.9	65.0; 60.0–80.0	56.6; 50.0–66.0	<0.001
BMI (Med; IQR)		22.6; 20.3–25.5	23.1; 20.8–25.8	22.1; 20.2–25.1	0.3
Education, n (%)					0.01
	Secondary	7 (6.8)	0 (0)	7 (10.2)	
	Technical school/diploma	20 (19.2)	3 (8.6)	17 (24.6)	
	University	77 (74.0)	32 (91.4)	45 (65.2)	
Organization, n (%)					0.51
	Public university	70 (67.3)	24 (68.6)	46 (66.7)	
	University hospital	34 (32.7)	11 (31.4)	23 (33.3)	0.92
Valid wearing day/week (M±SD)		6.6 ± 0.9	6.6 ± 1.0	6.5 ± 0.9	

BMI, body mass index; IQR, interquartile range; M, mean; Med, median; SD standard deviation.

^a^ Test of significant difference between males and females.

Fitbit Flex recorded a significantly higher (*P* < 0.001) number of daily step counts than that from the ActiGraph across all participants, by gender and each category of step-defined daily physical activity (Table 2). Males reflect significantly higher daily step counts from Fitbit Flex (*P* = 0.01) and ActiGraph (*P* = 0.028) compared to females.

**Table 2:** Comparison, relative agreement and median of absolute error in step counts between ActiGraph and Fitbit Flex: all participants, by gender and category of step-defined daily physical activity.

Step count/day	All (682 days)	Males (229 days)	Females (453 days)	Inactive (426 days)	Active (256 days)
Fitbit Flex (Med; IQR)	10193; 7490–12898^a^	11030; 7604–14838^a^	9992; 7397–12509^a^	8235; 6267–10003^a^	14075; 11948–16864^a^
ActiGraph (Med; IQR)	8812; 6152–11471^a^	9409; 6268–12897^a^	8599; 6053–11118^a^	6856; 4982–8465^a^	12716; 11112–14505^a^
Spearman’s rho	0.89*	0.91*	0.87*	0.76*	0.76*
ICC (95% CI)	0.85 (0.58–0.93)	0.87 (0.56–0.94)	0.83 (0.56–0.92)	0.73 (0.68–0.77)	0.82 (0.77–0.85)

CI, confidence interval; IQR, interquartile range.

^a^ ActiGraph and Fitbit Flex estimates are significantly different (*P* < 0.05).

* *P* < 0.01

The magnitude of the correlation and agreement in step counts between ActiGraph and Fitbit Flex were assessed (Table 2). Good to excellent significant positive correlations and agreement were shown in all participants, by gender and category of step-defined daily physical activity. Table 3 shows the number of days that were misclassified as active or inactive according to the Fitbit Flex. The proportion of overall agreement of devices in classifying days as active or inactive was estimated, reporting a kappa of 0.66, indicating a moderate agreement (Table 3).

**Table 3:** Agreement between ActiGraph and Fitbit Flex for categorizing step-defined daily physical activity.

No. of days (%)^a^	ActiGraph	
Fitbit Flex	Inactive	Active
Inactive	320 (46.9)	11 (1.6)
Active	106 (15.5)	245 (35.9)
Total	426 (62.5)	256 (37.5)
Kappa (95% CI)	0.66 (0.61–0.71)	

^a^ Physical activity categories are based on ActiGraph daily step counts: inactive <10,000 steps/day and active ≥10,000 steps/day [5].

**Fig 1:**
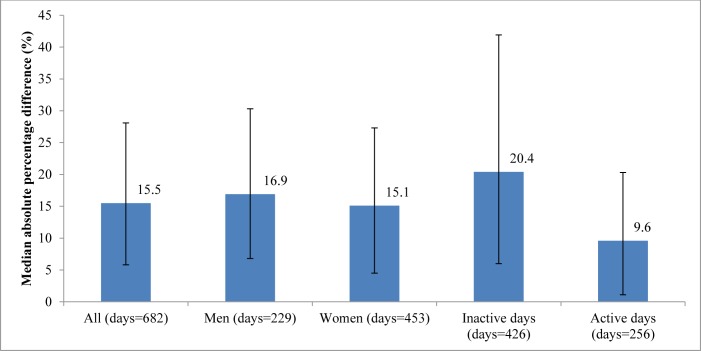
MAPE (%) between ActiGraph and Fitbit Flex. Error bars indicate IQR of MAPE. MAPE, median absolute percentage error.

Fig 1 shows the MAPE in number of steps between the two devices. Significant differences in the MAPE of step counts were found between devices across step-defined physical activity categories (*P*<0.001), but not for gender (*P* = 0.17).

**Fig 2:**
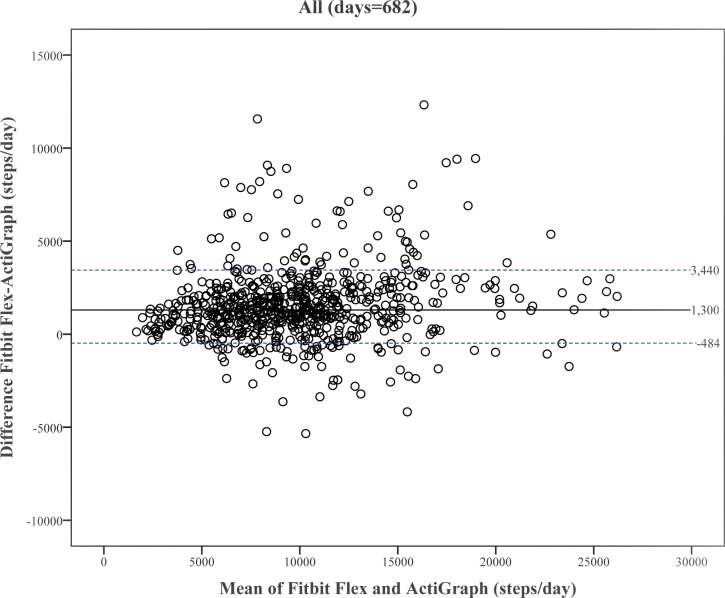
Bland-Altman plot of differences between waist-worn ActiGraph and wrist-worn Fitbit Flex against the mean according to all participants. The solid line represents median of the differences between devices, dotted lines are 10^th^ and 90^th^ percentiles of the differences.

**Fig 3:**
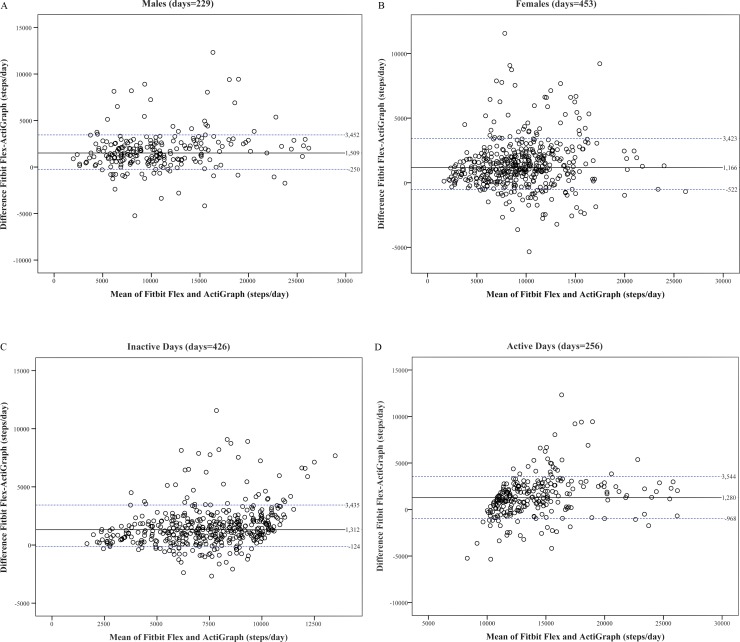
**Bland-Altman plots of differences between waist-worn ActiGraph and wrist-worn Fitbit Flex against the mean according to: (A) Males, (B) Females, (C) Inactive days, and (D) Active days.** The solid lines represent median of the differences between devices, dotted lines are 10^th^ and 90^th^ percentiles of the differences.

Figs 2 and 3A–3D present Bland-Altman plots on the median of differences, and the 10^th^ and 90^th^ percentiles between steps/day obtained from Fitbit Flex and ActiGraph. The bias (median difference) is 1300 steps/day for all participants. In general, the Fitbit Flex overestimated steps/day relative to ActiGraph (median differences range: 1166–1509 steps/day by gender and 1280–1312 by step-defined physical activity categories).

## Discussion

This study focused on the direct comparison of steps obtained from the Fitbit Flex and ActiGraph. The results show positive correlations and agreement in step counts of free-living adults as measured by the waist-worn ActiGraph and wrist-worn Fitbit Flex activity monitors. At the same time, overestimation of step counts and classification as active days by Fitbit Flex were found. This may have important public health implications if consumers or participants of health promotion programs are identified as being active when in fact they are not.

Recently, a number of studies have investigated the accuracy of various consumer-based physical activity trackers, recognizing the role they may play in physical activity promotion. For instance, Case et al. [16], Storm et al. [20], and Diaz et al. [21] have validated consumer wearables for measuring steps. However, to date very few studies have investigated the accuracy of these monitors under free-living conditions [24]. This is highly important because the accuracy of devices may differ considerably in day-to-day life as compared to under highly controlled and short protocols of activities. Recently, several studies have been conducted with regard to this important research question [25–27]. Dierker et al. [25] assessed the validity of Fitbit Flex among 17 college-aged adults and found that although the steps measured by Fitbit Flex (9596 ± 2361 steps) were higher than the ActiGraph GT3X+ (7766 ± 2388 steps), the difference was not statistically significant (*P* = 0.052). However, the authors instructed the participants to remove the devices while they were exercising over the 7-day monitoring period; hence it is possible that not all free-living movements have been captured as in the present study. In another study by Dominick et al. [26], the Fitbit Flex registered a total of 10286 ± 3760 free-living steps/day as compared to the ActiGraph of 9639 ± 3456 steps/day (albeit no significant difference was found between devices) among 19 participants. In contrast, Sushames et al. [27] reported a larger absolute difference of over 3000 steps (47.0%) in free-living steps between Fitbit Flex and ActiGraph among 25 adults, of which the Fitbit Flex has underestimated step counts. The reason for this underestimation from Fitbit Flex is unclear, but it could be related to the variability in participants’ movements or undercounting of steps by the Fitbit Flex.

Different study settings and reference methods could contribute to the discrepancies in outcomes. Kooiman et al. [39] assessed the validity of Fitbit Flex over 1 day in a smaller sample of free-living adults and found high agreements in steps with the activPAL. They found a noticeably smaller mean absolute percentage difference of 3.7% against the activPAL [39]. In accordance with our findings, another recent study comparing Fitbit Flex and ActiGraph on 48 cardiac patients (mean age: 65.5 years), in which high correlations and a difference in step counts of 1038 steps/day in the total population over 4 days of monitoring period were reported. Thus, comparing findings among different populations can provide an implication of how reproducible and valid this device is. It was also noted that the overestimation in step counts by the Fitbit Flex in this study resulted in a considerable misclassification of days as being active, which may have important public health implications. As shown in our analysis, the differences in steps between Fitbit Flex and ActiGraph were larger on inactive days as compared to active days.

Hypothetically, as most lifestyle activities include movements at the wrist, people might have performed movements such as hand waving that could be identified as potential false positive events/steps by Fitbit Flex. It was apparent that wrist-movements could reflect arm/forearm motions with a relatively small mass (while sitting), or they could be classified as step counts (while walking or running) [40]. Tudor-Locke et al. [35] found a large difference even using the same ActiGraph device placed between different attachment sites. They further reported that the difference between mean steps from the wrist and waist was 2558 steps under free-living conditions, with a higher average step counts on the wrist [35]. In line with this, Hilderbrand et al. [41] found a 200% higher step activity from the wrist-worn GENEActiv than the waist-worn ActiGraph in some adults. These observations suggest room for further progress, since recent studies reported using wrist-worn monitors resulted in improved wearing compliance due to comfort issues and without having the need to remove them intermittently [42]. Ultimately, prolonged wear time would improve data quality as the issue of missing data due to non-compliance could be minimized.

### Strengths

Despite the growing body of evidence, this study expands substantially on previous studies. Most importantly, as highlighted earlier, the comparison of the devices was done under free-living conditions for estimation of unstructured lifestyle activities. Secondly, the relationship between these devices were assessed for 7-day of wearing protocol. Thirdly, this study was conducted among a relatively large sample of adults. Fourthly, the performance of the devices was compared across different subgroups (males vs. females and step-defined physical activity categories).

### Limitations

This study may have limited generalizability as participants were predominantly females, relatively young and healthy. Furthermore, the use of ActiGraph as the reference instrument has its drawbacks. It is possible that the difference in steps between devices could be attributable to not only the Fitbit Flex, but also the ActiGraph, which is not the gold standard for measuring step counts [43]. However, the ActiGraph has been shown to be a valid tool to assess step count (as compared with the Omron pedometer and Yamax Digiwalker [11, 12]), and it is practical for use in epidemiological studies [44]. Careful consideration should also be given to the effects of movement artefact and signal noise due to the use of devices that are not attached directly to the skin (i.e. Fitbit Flex worn on a wrist-band and ActiGraph on a waist-belt), which might have affected the devices’ functionality to accurately measure step count. Being limited to only step count data, there was no indication as to whether the activities performed were of light-, moderate- or vigorous-intensity level. In general, step counts from accelerometers of different attachment sites (i.e. wrist- and waist-worn) might not be ideal for a direct comparison; nonetheless, results of this study were more likely to reflect the performances of these devices in real-world practice.

## Conclusions

Positive correlation and agreement in step counts were found between wrist-worn Fitbit Flex and waist-worn ActiGraph in free living adults, which is consistent with the existing evidence mainly from laboratory studies. However, a considerable overestimation of Fitbit Flex was noted, which resulted in substantial misclassification by Fitbit Flex when applying common step count recommendations. This can have important practical implications for the use of these devices by researchers, practitioners and health promoters, which often use the achievement of certain step count goals or increases in step counts as desired outcomes. Evidence presented in this paper adds to the existing literature on the validity of consumer devices for physical activity monitoring and these cautionary limitations should be considered in the design of study data collection and health promotion strategies.
